# Should We Consider Sacral Nerve Stimulation as a Treatment for Neurogenic Lower Urinary Tract Dysfunction? ICI‐RS 2025

**DOI:** 10.1002/nau.70181

**Published:** 2025-11-18

**Authors:** Marcus J. Drake, Mathijs de Rijk, Stefania Musco, Jalesh Panicker, Michał Późniak, Nikita Bhatt, Brigitte Schurch

**Affiliations:** ^1^ Department of Surgery and Cancer Imperial College London UK; ^2^ Department of Urology Charing Cross Hospital, Imperial College Healthcare Trust London UK; ^3^ Department of Urology, Mental Health and Neuroscience Research Institute, Faculty of Health, Medicine and Life Sciences Maastricht University Maastricht the Netherlands; ^4^ Department of Urology Maastricht University Medical Centre+ Maastricht the Netherlands; ^5^ Neuro‐Urology, Azienda Ospedialiero‐Universitaria Careggi Firenze Italy; ^6^ Department of Uro‐Neurology, The National Hospital for Neurology and Neurosurgery University College London NHS Foundation Trust London UK; ^7^ Department of Translational Neuroscience and Stroke, UCL Queen Square Institute of Neurology, Faculty of Brain Sciences University College London London UK; ^8^ Department of Urology and Andrology, Collegium Medicum Nicolaus Copernicus University Bydgoszcz Poland; ^9^ Urology department Newcastle Hospitals NHS Foundation Trust UK; ^10^ Neurourology Unit, Department of Neuroscience University Hospital Lausanne CHUV Lausanne Switzerland; ^11^ Neurourology Unit, Hirslanden Clinic Geneva Switzerland

**Keywords:** incontinence, neurogenic bladder, neuromodulation, sacral nerve stimulation

## Abstract

**Aims:**

To explore the boundary of clinical use of sacral nerve stimulation (SNS) in neurogenic lower urinary tract dysfunction (NLUTD), identifying barriers to approval and early‐impact research questions.

**Methods:**

This review is derived from a proposal discussion at the International Consultation on Incontinence‐Research Society in Bristol in June 2025.

**Results:**

Current evidence for predicting NLUTD symptom improvement or functional recovery after SNS remains mainly from small retrospective cohorts. Definitive trials are a research priority, accordingly. The influence of SNS on urodynamic parameters is uncertain due to the lack of studies with urodynamics as the primary endpoint. Additionally, there is no core outcome set for NLUTD treated with SNS. Use of closed‐loop SNS in NLUTD to adjust stimulation parameters may improve outcome and device longevity. Hence, we need to elucidate how SNS modulates LUT control network over time and across disease stages. Optimal stimulation parameters need to be defined for patient populations, but also for individuals, with dynamic strategies for adjustment. Use of SNS in NLUTD needs to enable ongoing use of MRI scanning for neurological evaluation, especially in progressive conditions. An MR Conditional SNS device means it can be safely scanned in specific MR environments. Use of such a device needs to factor in the range of further electronic implants that might be used in complex medical conditions.

**Conclusions:**

The application of SNS to NLUTD is likely to increase. A key aspiration is nuanced patient selection, using functional assessment and urodynamic findings. To translate SNS into standard clinical practice, interdisciplinary collaboration and robust clinical trials are essential.

## 1 Introduction

Beyond its US Food and Drug Administration (FDA)‐approved indications, sacral neuromodulation (SNS) has been increasingly explored in a variety of off‐label contexts for patients with neurologenic lower urinary tract dysfunction (NLUTD). Indeed, in 2024, the European Association of Urology guideline chose to position SNS as a treatment equivalent to intradetrusor botulinum toxin in NLUTD.

An evidence base is emerging for SNS in Parkinson's disease (PD), multiple sclerosis (MS), spina bifida, and the broader category of neurogenic bladder (NB). Despite this growing evidence base, SNS is not yet formally indicated for NLUTD. Barriers to FDA approval include the logistical and financial challenges of conducting large‐scale randomized controlled trials in heterogeneous populations, as well as the difficulty of defining uniform inclusion criteria and outcome measures across varying neurological etiologies. In fact, most studies were limited by small sample sizes, short follow‐up durations, and lack of stratification by disease severity—highlighting the need for larger, controlled studies to determine long‐term effectiveness and refine patient selection.

Nevertheless, the high prevalence of neurogenic bladder and the limitations of current therapies suggest a substantial unmet need. Expanding SNS indications in appropriately selected patients could improve symptom control, bladder function, and quality of life, while reducing risks for the upper urinary tract. Moving forward, collaborative multicenter studies with standardized protocols will be essential to validate SNS's efficacy, identify predictors of response, and inform evidence‐based guidelines. As our understanding of disease‐specific pathophysiology deepens, so too will opportunities to personalize neuromodulation therapies and expand access to this promising intervention for underserved patient populations.

As the need for MRI imaging becomes more common for diagnosis and disease surveillance, device selection based on MRI conditionality is more important. However, the decision to implant a particular device should still be driven primarily by selecting one that has the best opportunity to treat each individual patient's disease.

The current review is derived from a proposal discussion at the International Consultation on Incontinence‐Research Society in Bristol in June 2025, with a key aim to explore the boundary of clinical use to identify early‐impact research questions.

## 2 Background

In 1997, the FDA approved SNS for urgency urinary incontinence based on pivotal randomized studies [1, 2]. Since then, use has gradually broadened off‐licence to include NLUTD. Preclinical work showed that chronic S3 stimulation abolished detrusor hyperreflexia and attenuated C‐afferent neuropeptide upregulation in spinalised rats, implicating modulation of C‐fibre activity in the mechanism of action of SNS [3]. Clinically, Chartier‐Kastler et al. reported durable increases in bladder capacity and a reduction in incontinence episodes in nine women with drug‐refractory neurogenic detrusor overactivity, suggesting that SNS can serve as a reversible alternative to bladder augmentation [4]. The first observations in patients with complete spinal‐cord injury (SCI) were published by Schurch et al. in 2003 [5]. Building on this concept, Sievert et al. implanted an S3 lead within 6 weeks of acute SCI and demonstrated that early “preventive” neuromodulation maintained low‐pressure storage and avoided the development of detrusor over‐activity in most participants [6].

Disease‐specific series confirmed efficacy in MS. Minardi et al. reported that, after two‐stage screening, 15 of 25 MS patients received a permanent generator, and 66% responded over a mean follow‐up of 5 years [7]. Greenberg et al. documented approximately 60% symptom improvement in a cohort with PD [8]. The introduction of full‐body MRI‐conditional leads in 2019 eliminated a key contraindication for neurological populations requiring serial imaging. Incremental hardware refinements, such as tined leads and mini‐implantable pulse generators (IPGs), are also now available.

## 3 Knowledge Base Gaps

Current evidence for predicting functional recovery after sacral neuromodulation remains limited to small studies, based mainly on retrospective cohorts, but a few phenotypic signals are emerging. In patients with detrusor underactivity (DU), the best responders tend to be younger and to have some residual contractility on baseline pressure‐flow studies [9]. Sex does not appear to influence outcome, as female and male patients improve at comparable rates [10]. In detrusor–sphincter dyssynergia (DSD), neuromodulation consistently decreases dyssynergia episodes. A dyssynergic bladder neck–detrusor subtype (DBND) may have the highest likelihood of resolution with SNS [11]. Suprapontine injuries fare worse. Sacral or conus lesions with preserved afferent integrity retain the greatest capacity for restoration of coordinated voiding.

The effect of SNS on urodynamic parameters remains unclear, because there are few studies where urodynamics is the main focus. There is also no universal agreement on which urodynamic parameters should be included in clinical trials, such as maximum cystometric capacity, end‐fill detrusor pressure, bladder compliance, and post‐void residual. Standardizing these variables would help combine data more effectively and clarify how SNS influences NLUTD.

Static versus progressive neurological disease has implications for therapy. Early timing seems crucial in acquired static lesions. In acute spinal shock, S3 implantation within 6 weeks prevented neurogenic detrusor overactivity in 78% of patients [6]. In contrast, implantation years after injury mainly yields symptomatic relief and is less likely to reverse established fibrosis [12]. Which chronic SCI phenotypes conceivably might benefit once irreversible bladder change has occurred remains uncertain. For progressive disorders, a systematic review of SNS in MS found response rates of 51–83%, with greater benefit in patients with mainly storage symptoms [13]. In PD, a small series reported 70% clinical improvement [14]. Nevertheless, the evidence base remains weak: there is no ICS core outcome set for NLUTD treated with SNS and no randomised trials, pending outcome of the sham‐controlled NEMISIS study [15].

SNS has not been widely researched in neurological disorders affecting the peripheral nervous system [16]. One study following SNS in patients with non‐obstructive urinary retention suggests that patients with disorders affecting lower motor neurones have a worse outcome [17]. However, the type of neurological disorder and the extent of conus and sacral root were not analysed. Ambulatory patients with spina bifida may experience improved continence and urodynamic parameters (e.g., bladder compliance and detrusor overactivity) with SNS [18]. In this situation, patient selection should extend beyond urological parameters and consider the variability in sacral anatomy due to the dysraphism and the extent of involvement of the sacral roots. Pelvic neurophysiological tests, including pudendal sensory evoked potentials, bulbocavernosus reflex, and anal sphincter electromyography (EMG), can evaluate the somatic sensory and motor innervation from sacral S2‐S4 roots [19]. These tests could be used to phenotype the extent of sacral root involvement neurologically in patients with spina bifida being considered for SNS.

## 4 Stimulation Parameter Optimization and Closed‐Loop Strategies

SNS is indicated to influence bladder function primarily via specific sacral nerve stimulation (SNS) frequencies and patterns. Animal research suggests that variations in parameters such as frequency, pulse width, and amplitude can differentially affect bladder activity, capacity, and external urethral sphincter function [20]. Nonetheless, the underlying neurophysiological pathways remain poorly characterized, and it is unclear whether particular stimulation parameters selectively target discrete components of the LUT control network or produce broader modulatory effects across multiple nodes. Critical knowledge gaps include whether effective stimulation frequencies are consistent across patient populations and disease stages, and how they may affect a nervous system that is undergoing progressive degeneration. Disease‐associated changes over time, such as changes in sensory thresholds, reflex circuitry, or network excitability, further complicate the identification of durable, phenotype‐ or patient‐specific stimulation paradigms. This underscores the potential of closed‐loop systems, which are capable of adapting in real‐time based on physiological feedback.

At the supraspinal level, the coordination of storage and voiding is orchestrated by brainstem circuits, particularly the pontine micturition center (PMC) and the periaqueductal gray (PAG), which act as central “switches” integrating afferent information from the LUT [21]. The effects of peripheral interventions, such as SNS, on these central nodes remain largely unknown. Key questions include whether SNS restores normal switching dynamics, bypasses damaged pathways, or engages alternative circuits. Understanding these interactions is essential for the development of closed‐loop neuromodulation systems, which require robust markers to guide precise and timely stimulation. Neuroimaging studies have shown that SNS can modulate PAG activity, effectively raising the “set‐level” at which the PAG responds [22]. These findings suggest the possibility of tailoring stimulation protocols to central circuit dynamics. Additional questions relate to network reorganization in NLUTD, especially in progressive conditions, and the identification of optimal feedback sites for adaptive control.

Conditional neuromodulation has yet to be widely applied to bladder control devices, but equivalent technology is applied in cardiac pacemakers to provide real‐time, responsive adjustments [23]. For SNS, the concept involves using implantable sensors, such as pressure sensors within the bladder or cuff electrodes on afferent nerve roots, to provide feedback to the IPG. This feedback mechanism allows the device to respond dynamically to the body signals. Closed‐loop neuromodulation combining simultaneous recording and stimulation with real‐time parameter adjustment, has been explored in preclinical models using detrusor or dorsal root ganglion activity to trigger adaptive interventions [24, 25]. Early human systems typically record and stimulate at the sacral level. However, the clinical utility of the recorded signals is uncertain, as the relationship between sacral neural activity and LUT activity remains poorly understood. A recent study using urodynamics as input for adaptive stimulation demonstrates that automated closed‐loop stimulation, triggered by an algorithm detecting bladder contractions during urodynamics, can inhibit neurogenic bladder overactivity [26]. This highlights the therapeutic potential of real‐time closed‐loop neuromodulation. Translating these approaches to clinical practice requires identification of reliable biomarkers that reflect both normal and pathological LUT control, alongside development of algorithms capable of dynamically adjusting stimulation parameters in response to changes in neural activity and disease progression. This challenge is of particular importance in progressive neurogenic conditions, where static stimulation paradigms may lose efficacy or become counterproductive over time. Longitudinal studies that capture neural signals alongside functional outcomes are therefore critical, as is understanding whether adaptive systems can anticipate the need for parameter adjustments or must wait for functional decline before recalibration.

Three overarching priorities are central to advancing closed‐loop SNS in NLUTD. First, elucidating how SNS modulates the LUT control network over time and across disease stages. Second, defining optimal stimulation parameters not only for patient populations but for individuals, with dynamic strategies for adjustment. Third, integrating this mechanistic understanding into closed‐loop systems that are adaptive and patient‐specific. Addressing these questions is essential to realizing the full potential of neuromodulation as a safe, effective, and personalized therapy for neurogenic patients.

## 5 Device Safety and MRI Image Quality

**Figure 1:**
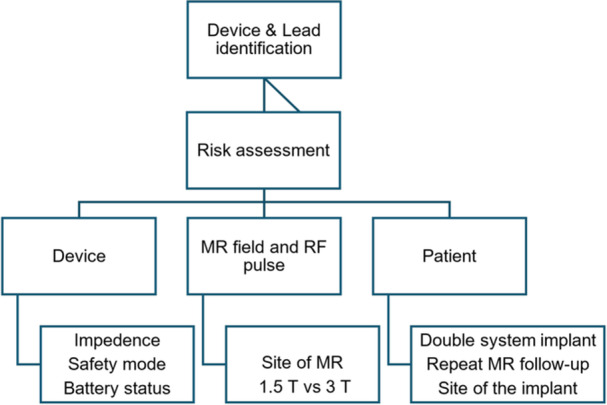
SNS patient safety and imaging quality considerations for MRI.

As defined by the American Society for Testing and Materials (ASTM) standard, a device can be classified as MR Safe, MR Conditional, or MR Unsafe based on its interaction with an MRI scanner. An MR Conditional SNS device means the device can be safely scanned in certain and specific MR environments [27]. Specifically, only the fully implanted permanent system is eligible and needs to be intact, located within the sacral foramen and with a certain amount of residual battery. Moreover, the prevalence of neurological patients with electronic implants for their primary disease (e.g., deep brain stimulation), related issues (e.g. epilepsy, neurogenic LUTD, spasticity, neuropathic pain) or other comorbidities (e.g. cardiac pacemakers) underscores the critical need for standardized MR safety protocols to reduce the risk of MR‐related accidents. Hence, to ensure patient safety and imaging quality, several factors related to the device, the MR field and radiofrequency (RF) pulse, and the patient's characteristics should be considered (see Figure 1).

According to the manufacturers, only newer SNS models are full‐body 3 T MR conditional. If a patient has an abandoned lead fragment smaller than 6 cm, 1.5 T MR scanning could still be feasible under specific restrictions. Potential risks in patients with abandoned leads include RF‐induced heating, alteration of capture threshold, and discomfort [28, 29]. This raises some legal issues regarding the length and the type of electrode implanted, since X‐ray markers to discriminate the model are present only on the IGP, not on the electrode [27, 30].

Current SNS systems allow a continuous MR scan for not longer than 30 minutes with different specific absorption rate (SAR) and RF thresholds as labelled by the manufacturers. It is not clear how these limitations may affect the quality of the imaging. Moreover, there is no data regarding the magnetic susceptibility and risk of artifacts which may occur when MR is done near the SNS device, such as image distortions/propagations or signal loss [30, 31]. However, some authors found that patients with non‐conditional SNS devices showed no serious adverse effects when MR was performed at 1.5 T, including in the lumbar zone [32].

Considering the importance of MR as a diagnostic tool for the diagnosis and follow‐up of neurological disease, ultrahigh‐field MR at a field strength of 7 T has been progressing. Hence, the implications of imaging with MR scanners higher than 3 T should be considered with the patient [33, 34]. Literature on any MR‐conditional neuromodulation devices and pacemakers reports several consequences for the device, such as a battery voltage reduction, parameters or impedance change, electrical reset, increased threshold and altered sensation. Many can be identified and resolved, but what is still unclear is the potential cumulative effects of repeated MR scans. Furthermore, no data are available regarding the effects on MR in patients in possession of more than one stimulation or equivalent device [35].

Hence, rapid evolution of neuromodulation systems and its application in neuro‐urological patients, a best practice approach and design standardized protocols are needed for safety and best MR performance. This includes in non‐conditional or conditional SNS, planning for feasibility in ultrahigh‐field conditions [35, 36].

A key issue is the classification of devices is affected by the extent to which their manufacturers have tested the MR safety, which potentially differs from the extent to which the device is actually MR safe. Devices can mostly withstand more than they are approved for; however, manufacturers may adopt a cautious approach and not approve more MR exposure than absolutely necessary.

## 6 SNS in Neuropaths – What Next?

In a limited number of cases, closed‐loop methods have been tested in individuals with SCI. The strategy involved stimulating the dorsal genital nerve only when bladder filling pressures or detrusor contractions exceeded a predetermined threshold (8–12 cm H_2_O above baseline) [23]. This method resulted in some improvement in bladder capacity when compared to continuous stimulation protocols. Unlike the fixed stimulation protocols, adaptive systems have the potential to improve long‐term efficacy and reduce therapy failure by tailoring treatment to individual bladder activity patterns as they evolve. While promising, this remains an area that requires more research and development [23, 37].

Applying SNS in myelomeningocele patients is hampered by anatomical variations, resulting in difficulty in localisation of the sacral foramina. Imaging techniques might help guide the procedure in such cases, for example, using 3D imaging such as the O‐arm for intraoperative fluoroscopy to aid in locating the foramina [38]. Fusion images of sacrococcygeal CT and MR sacral plexus nerve images using a 3D Slicer have been used as a reference [39]. The 3D map provided can aid in preoperative evaluation and intraoperative implantation, improving targeting of the nerve roots.

Advances in device design and remote monitoring may serve to expand access, improve long‐term outcomes and optimise patient‐centred care in this population. Development of minimally invasive and wearable neuromodulation systems could broaden access for patients who are not candidates for current implants [40]. Furthermore, improvements in battery technology, such as longer‐lasting or rechargeable IPGs, promise to enhance device longevity and reduce the need for repeat operations. Finally, the integration of telemedicine and remote monitoring will allow clinicians to adjust therapy parameters in real time more efficiently, ultimately improving patient adherence and access to care, especially for those in remote areas [40].

## 7 Further Research Questions

- Which LUTS domain (storage vs voiding) best predicts response?
- How should treatment timing be tailored in a changing neurological condition, such as relapsing‐remitting versus progressive MS?
- Does SNS have a role in NLUTD due to disease of higher centres, such as stroke, dementia or multiple system atrophy?
- Is there a group of patients with peripheral nerve disease who could benefit from SNS?
- Can pelvic neurophysiology testing identify a cohort of patients with spina bifida who may respond to SNS?
- In slim patients or those with atypical S3 anatomy, does antegrade sacral‐root lead placement reduce migration and improve pain control compared with the standard retrograde technique?
- In patients at risk of renal dysfunction due to NLUTD, can SNS lower end‐fill pressure and protect renal function?
- Does bilateral SNS restore efficacy after unilateral failure?
- How does repeated MRI scanning affect patient safety and the long‐term functional integrity of SNS systems?
- How can SNS system technology be improved to enhance MRI safety and feasibility, particularly in ultrahigh‐field MRI settings?
- What standardized MRI protocols can be designed to ensure optimal imaging performance and diagnostic accuracy in neuro‐urological patients with SNS implants?
- How can SNS dynamically modulate LUT control across disease stages, and how can patient‐specific, adaptive stimulation strategies be optimized for individualized, closed‐loop therapy?

Most of these questions would be best suited to a cohort or case‐control design as there is insufficient data on which to base a design or a randomised controlled trial.

## 8 Conclusions

With the advent of the MRI‐compatible devices, the application of SNS to NLUTD is likely to increase. The current evidence has several limitations, including small studies with retrospective design, selection bias, the heterogeneity of the population being studied and the lack of longer‐term follow‐up [41]. With the rapid emergence of new neuromodulation devices and advanced MRI imaging, consensus guidelines, exemplified by the one provided by the American Society of Pain and Neuroscience (ASPN) [42], can play an important role in patient safety.

A key aspiration is nuanced patient selection, which should be based on a thorough functional assessment and urodynamic findings rather than solely on a neurological diagnosis. Identifying the optimal candidate is crucial for maximizing treatment success. To translate newer developments in SNS for nLUTD into standard clinical practice and achieve broader adoption, interdisciplinary collaboration and robust clinical trials will be essential.

## Ethics Statement

The authors have nothing to report.

## Consent

The authors have nothing to report.

## Conflicts of Interest

MD; Trustee of the International Continence Society, Speaker/advisory board for Astellas, Viatris. MdeR; none. SM; Speaker/advisory board for Hollister Italia, Convatec Italia, Teleflex Italia, Bbraun Italia, Ecupharma, Pierre‐Fabre Italia, Pierre‐Fabre Medicament (France). JP none relevant. MP; none; NB; none. BS; none relevant.

## Data Availability

Data sharing is not applicable to this article as no datasets were generated or analysed during the current study.
